# An Overview on the Therapeutic Function of Foods Enriched with Plant Sterols in Diabetes Management

**DOI:** 10.3390/antiox10121903

**Published:** 2021-11-27

**Authors:** Selvaraj Jayaraman, Anitha Roy, Srinivasan Vengadassalapathy, Ramya Sekar, Vishnu Priya Veeraraghavan, Ponnulakshmi Rajagopal, Gayathri Rengasamy, Raktim Mukherjee, Durairaj Sekar, Reji Manjunathan

**Affiliations:** 1 Department of Biochemistry, Saveetha Dental College & Hospitals, Saveetha Institute of Medical & Technical Sciences, Chennai 600077, India; vishnupriya@saveetha.com (V.P.V.); gayathri.sdc@saveetha.com (G.R.); 2 Department of Pharmacology, Saveetha Dental College & Hospitals, Saveetha Institute of Medical & Technical Sciences, Chennai 600077, India; anitharoy@saveetha.com; 3 Department of Pharmacology, Saveetha Medical College and Hospital, Saveetha Institute of Medical & Technical Sciences, Chennai 602105, India; srinivasanv.smc@saveetha.com; 4 Department of Oral Pathology, Meenakshi Ammal Dental College and Hospitals, Chennai 600095, India; drramya.oralpathology@madch.edu.in; 5 Department of Central Research Laboratory, Meenakshi Ammal Dental College and Hospitals, Chennai 600095, India; ramgslaks@gmail.com; 6 Shree PM Patel Institute of PG Studies and Research in Science, Sardar Patel University, Anand 388001, India; raktimmukherjee2003@gmail.com; 7 Centre for Cellular and Molecular Research, Saveetha Dental College and Hospitals, Saveetha Institute of Medical & Technical Sciences (SIMATS), Saveetha University, Chennai 600077, India; 8 Multi-Disciplinary Research Unit, Chengalpattu Government Medical College, Chengalpattu 60300, India

**Keywords:** diabetes, insulin resistance, plant sterols, enriched foods, epigenetics, inflammation, therapeutic implications

## Abstract

Diabetes is one of the most significant health issues across the world. People identified with diabetes are more vulnerable to various infections and are at a greater risk of developing cardiovascular diseases. The plant-based food we consume often contains many sterol-based bioactive compounds. It is well documented that these compounds could effectively manage the processes of insulin metabolism and cholesterol regulation. Insulin resistance followed by hyperglycemia often results in oxidative stress level enhancement and increased reactive oxygen species production. At the molecular level, these changes induce apoptosis in pancreatic cells and hence lead to insulin insufficiency. Studies have proved that plant sterols can lower inflammatory and oxidative stress damage connected with DNA repair mechanisms. The effective forms of phyto compounds are polyphenols, terpenoids, and thiols abundant in vegetables, fruits, nuts, and seeds. The available conventional drug-based therapies for the prevention and management of diabetes are time-consuming, costly, and with life-threatening side effects. Thereby, the therapeutic management of diabetes with plant sterols available in our daily diet is highly welcome as there are no side effects. This review intends to offer an overview of the present scenario of the anti-diabetic compounds from food ingredients towards the therapeutic beneficial against diabetes.

## 1. Introduction

Diabetes mellitus (DM), commonly known as diabetes, is an endocrine disorder characterized by elevated glucose levels [1]. The pancreatic cells produce a metabolic peptide hormone called insulin which allows the entry of glucose from the blood to the cells to support them with energy. A lack of adequate insulin functional balance plays a vital role in diabetes development [2]. Diabetes is a chronic disease, and its global prevalence is estimated to be 4.4% in 2030. According to the International Diabetes Federation (IDF) data, it is estimated that by the year 2045, about 693 million people will be affected by diabetes worldwide, and the most badly hit countries will be the USA, China, and India [3]. Diet plays a significant role in the etiology of many diseases such as diabetes, cancer, and cardiovascular illness, even at a younger age. A healthy diet pattern (less salt, sugars, saturated, and industrial trans-fat but with more green and leafy vegetables and fruits) is essential to maintain good health and to prevent many diseases. Having the habit of following an unhealthy and uncontrolled diet is considered one of the fundamental reasons behind the induction of obesity and overweight [4]. The overweight and obese conditions can lead to many unhealthy situations such as cardiovascular diseases, muscular-skeletal disorders, and certain cancers associated with breast, ovarian, colon, and liver. At present, the epidemic death proportion due to obesity-related diseases has reached at least 2.8 million deaths per year globally [5].

Abnormal accumulation of fats results in insulin resistance, impaired glucose tolerance, and even diabetes [6]. In obese conditions, the level of major inflammatory proteins such as interleukin-6 (IL-6) and tumor necrosis factor (TNF-α) increases significantly. Elevated levels of these inflammatory markers induce dyslipidemia by inviting macrophages towards the adipose tissue [6,7]. Various conditions such as programmed cell death in pancreatic β cells due to insulin resistance, hyperglycemia-induced oxidative stress, and enhanced production of reactive oxygen species can lead to diabetes. The phytosterols are natural compounds that include sterol and stanol esters and are abundantly occur in plants’ cell membranes. The plant sterols that are commonly present in daily food intake are the β-sitosterol, campesterol, and stigmasterol. Due to the structural similarity with the body’s cholesterol, the phytosterols compete with the cholesterol and help in reducing the absorption of dietary cholesterol [8]. The European Foods Safety Authority (EFSA) declared that the daily consumption of plant sterols within the therapeutic dose (1.5–2.4 g/day) is adequate for disease prevention in humans without harmful side effects [9].

## 2. Diabetes

The Egyptian manuscript mentioned the diabetic condition as “too great emptying of urine”, dating back to 1500 BC. The great ancient Indian physician, Sushruta, and the surgeon Charaka identified two types of diabetic conditions at 400 AD, later named Type 1 and 2 diabetes [10]. The term “diabetes” was first introduced by Araetus of Cappodocia in 81–133 AD. Later, the word mellitus was coined by Thomas Willis in 1675 after finding the sweetness in the urine and blood of infected patients. Diabetes is a Greek word meaning “to syphon”, which indicates rapid drainage of fluid, and the word “mellitus” is a Latin word meaning honey [11]. The diabetic condition is classified into Type 1 and Type 2 diabetes, based on their etiology. Type 1 Mellitus is a chronic autoimmune condition in which the β cells of the pancreas stop the production of insulin because of autoimmune disorders and is more prevalent in adolescence. Patients with Type 1 diabetes appear thin to normal by body mass but are identified with ketoacidosis and absolute insulin deficiency. Administration of insulin is the treatment of choice for Type 1 diabetic patients [10].

On the other hand, the prevalence of Type 2 diabetes is more common than Type 1. People who are overweight or obese, 45 years or older, have a family history of diabetes, have pre-diabetic conditions, and are less physically active are more prone to be affected with Type 2 diabetes. Recent trends show that Type 2 diabetes is more common among young adults due to sedimentary lifestyles. Resistance to insulin results in elevated blood glucose levels and induces more insulin production by the pancreas. The work burden damages the β-cells of the pancreas and slowly stops the production of insulin. If unnoticed, the condition may badly affect the function of many organs such as the heart, nerves, blood vessels, eyes, and kidneys. There is no permanent cure for diabetes, but it can manage with medication and a healthy lifestyle [12].

### 2.1. Epidemics and Thrifty Genes

Diabetes is a global epidemic and will be the seventh leading cause of death by 2030. The island of Nauru, located in the Pacific Ocean, has the highest prevalence of type 2 diabetes globally, with more than 40% of people affected [13]. In the early 20th century, the Nauru people suffered from starvation due to lacking basic nutrition sustenance. In 1922, they discovered phosphate rocks in their land and were given royalties for the same. This incident made them rich, and people started leading an unhealthy sedentary lifestyle. They stopped agriculture and fishery farms, which resulted in the explosion of Type 2 diabetes. An American geneticist, Dr. James V. Neel, first proposed the “thrifty gene” concept. The theory explains that particular gene variants evolved to favor efficient use of nutrients in a calorie-limited environment may promote obesity and Type 2 diabetes under a modern calorie-rich environment. The thrifty genes potentially enable the collection and processing of food to deposit as fat during abundance to cope with periods of famine. In the case of the Nauruans, the thrifty genes might have been involved in the development of obesity and resulted in the massive epidemic outbreak of Type 2 diabetes [14].

### 2.2. Blood Glucose Regulation

**Figure 1:**
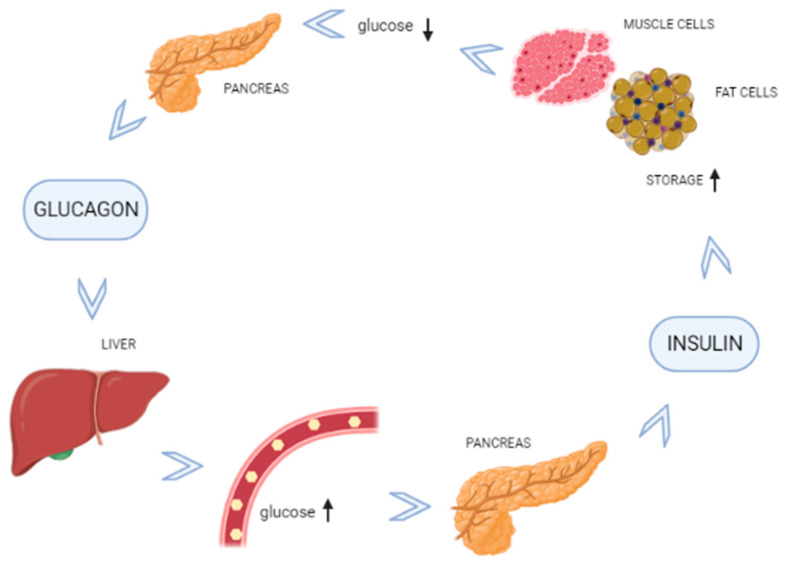
Physiology in the homeostasis of blood glucose level.

Glucose, the body’s primary energy source, can be used as fuel immediately or can be preserved in the form of glycogen in the liver and muscles. The blood glucose level increases steeply after a meal, and the enzymes insulin and glucagon regulate the elevated glucose level. The other hormones, such as somatostatin and amylin, also regulate glucose levels. Insulin is released when there is a surge of glucose in the blood to favor glucose absorption by somatic cells. Insulin also initiates glucose breakdown and helps in extra glucose storage in the liver as glycogen. Cells require insulin to support with protein synthesis process. Insulin can activate the components of the translational machinery, including elFs (eukaryotic initiation factors) and eEFs (eukaryotic elongation factors). In the long term, insulin increases the cellular content of ribosomes to augment the capacity for protein synthesis [15]. While the hormone glucagon performs the way opposite of insulin action (Figure 1).

## 3. Phytosterols

Phytosterols (PS) are plant-based sterols and stanols having a similar structure to cholesterol. They serve as a structural part of the plant’s biological membrane and are abundantly present in all plant-based food that we consume daily [16]. PS provides taste and aroma and chemically acts as an anti-oxidant, stabilizer, and modest radical scavenger. The most commonly available plant sterols are β-sitosterol, campesterol, and stigmasterol. They are majorly known for their hypocholesterolemic, immunomodulation, anti-diabetic, and anti-inflammatory potentials and play an essential role in DNA repair mechanisms [17].

Three significant forms of PS that are well explored up-to-date are:

- Polyphenols include flavonoids, phenolic acids, tannins, and stilbenes.
- Terpenoids include carotenoids and non-carotenoids.
- Thiols includes glucosinolates and indoles (Table 1).

### 3.1. Phytosterols Chemical Structure and Bioavailability

**Figure 2:**
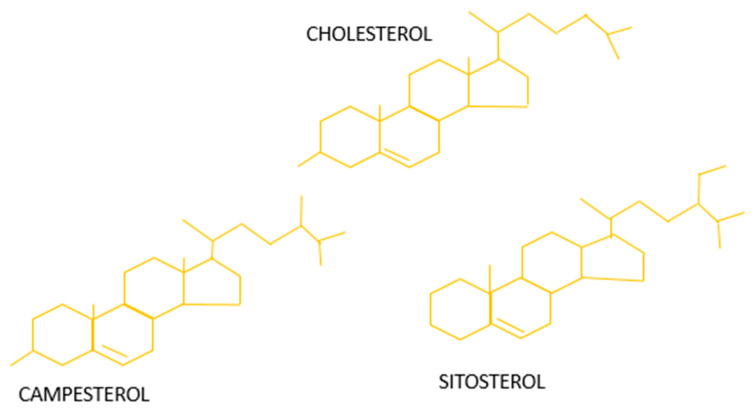
Structure of phytosterols.

Phytosterols have a characteristic structure. They have 17 carbon atoms arranged as four tetracyclic cyclopenta-α-phenanthrene rings with a C-24 atom side chain. Though they have structural similarity with cholesterol, they vary by a side chain of methyl or ethyl groups (Figure 2) [23]. Because of their lipid modulation properties, plant sterols have recently gained much attention [24,25]. Humans are naturally supplemented with plant sterols through the daily diet enriched with vegetables, fruits, wheat germ, whole grains, and many vegetable oils [26]. Plant sterols are present in two forms: free form (free sterols, FSs) and conjugated form [27]. Conjugated forms include: (1) steryl esters (SEs)— formed by coupling with higher fatty acids; (2) steryl glycosides (SGs)— formed by connecting with carbohydrates; and (3) acyl steryl glycosides (ASGs)—an acylated form of steryl glycosides. Suppose the PS consists of a methyl group at the 24th position; it forms a campesterol or can become a β-Sitosterol or stigmasterol if the position is replaced with an ethyl group. These components are present in the biomembranes of plants and play a vital role in structural stability, regulatory function, and signal transduction.

Compared to cholesterol, plant sterols are generally less permeable. Plant sterols are solubilized in bile salts and form micelles similar to cholesterol during absorption [28]. The absorption rate of plant sterols is about 5%, but it is 40 to 60% for cholesterol. The differential plant sterols exhibit different absorption rates, depending on the sterol nucleus and the side chain. The concentration level of sterols in plasma decreases from 0.1 to 0.14% based on the absorption rate [29]. Dietary supplementation of sterols reduces cholesterol absorption, lowers plasma LDL cholesterol levels, and functions as potent anti-oxidants. The plant sterols could modulate the immune response, oxidative stress, gut dysbiosis, mitochondrial dysfunction, and dyslipidemia. Studies have also reported that the sterols and stanols significantly regulate LDL-C serum levels [30]. Many in vitro examinations have been conducted to improve the bioavailability of plant sterols. The mutations that are identified in genes such as ABCG5, ABCG8, NPC1L1, and apolipoprotein E might result in the altered bioavailability of plant sterols [31]. Plant sterols are used therapeutically to lower the cholesterol level and in the formulations of many vitamins [32].

### 3.2. Mechanism of Action

**Figure 3:**
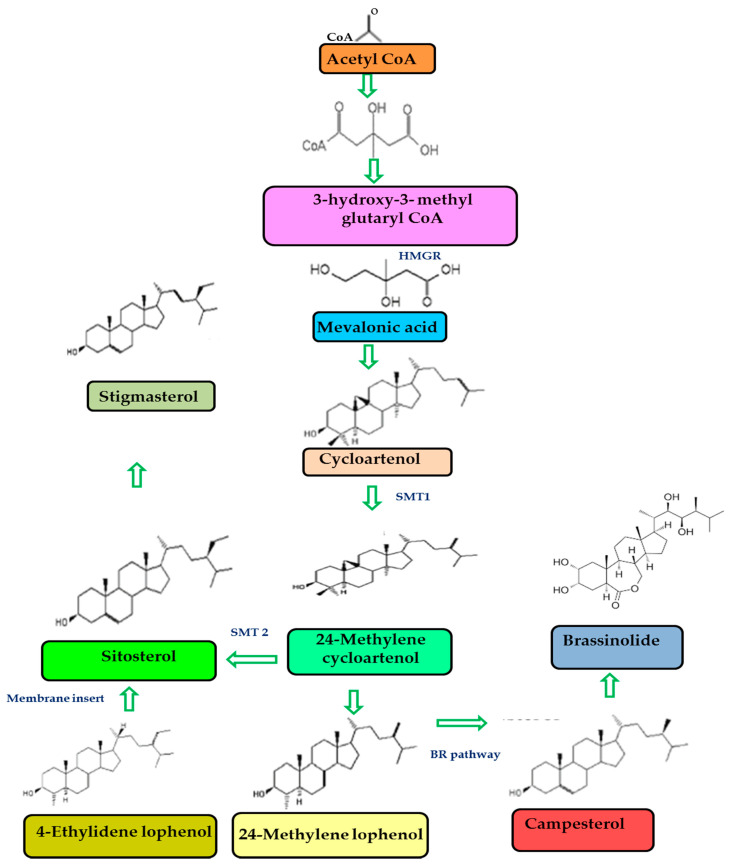
Schematic representation of biosynthetic pathway activated by the major plant sterols: HMGR-3-hydroxy, 3-methylglutaryl coenzyme A reductase; SMT-sterol 24-C-methyltransferase; BR-brassinosteroids.

Sterol formation is a multistage process and takes place in the endoplasmic reticulum. Initially, the acetate is converted into the squalene through C5-isoprene via the mevalonate pathway. Secondly, the cyclization of squalene happens and results in the formation of cycloartenol. The cycloartenol converts into the campesterol, β-sitosterol, stigmasterol, or brassicasterol based on ethyl or methyl groups addition [33] (Figure 3).

The bioavailability of these sterols has been studied by many researchers using both animal and human models [34]. Studies show that the body can absorb only about 5% of the plant sterols. In infants and young adults, the absorption rate seems to be higher than in adults [35]. Early studies reported that the absorption pattern of PS was a passive diffusion process. Later, it was shown that the sterols competitively solubilize in mixed micelles and cholesterol in the intestine [36]. A complex metabolic process occurs when the PS are taken up with the dietary cholesterol. These complex molecules are reduced into free sterols by the esterases and are taken by micelles. Micelles are the complex of bile salts, phospholipids, free sterols, and fatty acids. In comparison to cholesterol, plant sterols are more favorably solubilized in the intestinal lumen than cholesterol. The mixed micelles are taken up by enterocytes mediated by NPC1L1 at the brush border membrane. This absorbed cholesterol is esterified by fatty acids (ACAT-2) and incorporated into chylomicrons. The formed chylomicrons are first transported into the lymphatic vessel that penetrates each villus called the central lacteal. The chylomicron-rich lymph then drains into the lymphatic system and rapidly flows into blood. The blood-borne chylomicrons are rapidly disassembled, and their constituent lipids are utilized throughout the body. During the metabolic process of esterified cholesterol, the lipoprotein lipase acts upon the chylomicrons at the capillary bed and delivers triglycerides and fat-soluble vitamins to the tissues. The sterols in chylomicrons are primarily taken up by the liver and then cleared eventually. Excess un-esterified cholesterol is shuttle back to the intestine [37,38,39,40,41]. Numerous genes such as ATP binding cassette transporter (ABCG5/8) are involved in the secretion of un-esterified cholesterol. The plant sterol’s hypocholesterolemic effect depends on the induction of LXR and is regulated by NPC1L1 and ABC transporters [42,43,44,45,46]. However, the exact mechanism behind plant sterol’s absorption and transportation is still controversial. Due to the variation in ABCG5 or ABCG8, every individual shows a differential absorption pattern of PS [47]. Animal studies conducted by various researchers show that the plant sterols are highly distributed in the liver, adrenal cortex, ovary, and testis [46,48]. The half-life period of PS is 37, 17, and 15 min for campesterol, sitosterol, and stigmasterol, respectively. The plant sterols are usually not retained in the body, and most of them are excreted through bile in the feces [49]. The excretion of PS directly facilitates cholesterol excretion from the body and thereby aid in the maintenance of the hypocholesterolemic effect [47].

## 4. Diabetes and Cholesterol

Diabetes Mellitus, a standard, rapidly developing metabolic disorder affecting all age groups, are in serious need of medical attention [50]. By 2035, about 592 million people were expected to be affected by this disorder globally [51]. Diabetes is commonly associated with obesity, resulting in cardiovascular diseases such as ischemic heart disease and stroke [52]. Dyslipidemia is one of the most common features associated with obesity. It is also associated with Type 2 diabetes and is marked by increased triglycerides and lowered high-density lipoprotein levels. Type 1 diabetes is also observed with elevated triglycerides but with normal high-density lipoprotein levels. It has been observed that all the lipoprotein levels are found altered in diabetes due to hyperglycemia or insulin resistance [53]. Abnormal lipid profile is a strong indicator for coronary heart disease in Type 1 and 2 diabetic conditions. Although triglycerides do not participate directly in atherosclerotic plaque formation, they play a central role in constructing low-density lipids and impaired HDL cholesterol levels in diabetes conditions [54]. These changes, along with unaltered apoB and cholesterol levels in diabetes, lead to atherogenesis. Pieces of evidence show that therapies used in lowering cholesterol reduce the mortality rate due to cardiovascular diseases with or without diabetes.

### 4.1. Genetic and Epigenetic Modulations in Diabetes

Genetics plays a vital role in the development of Type 2 diabetes than Type 1. The same also has a more critical link to family history and lineage compared to Type I. Any mutations in proteins such as TCF7L2, ABCC8, GLUT2, and CAPN10 favor the progression of Type 2 diabetes because they could influence the production or mechanism of action of insulin (6). The other genes strongly linked with diabetes are class II MHC, HLA-DQ, HLA-DR, and HLA-DP proteins. Loss of insulin sensitivity in tissues, such as fat, muscle, and liver, is the cause of Type 2 diabetes development [7].

Moreover, Type 2 diabetes can be heritable and associated with obesity rather than its disease [55]. Thereby obesity forms the leading platform in the incidence and progression of DM. Many genes involved in this metabolic process are identified through genome-wide association studies (GWAS), candidate gene studies, and linkage studies. The significant genes associated with T2DM include CAPN10 belonging to the calpain family, involve intracellular remodeling and signaling activities [56,57]. The TCF7L2 gene is the most consistently applied in T2DM induction and produces a transcription factor that affects the Wnt signaling pathway. Many experiments have proven that the increased secretion of TCF7L2 results in the lowering of insulin secretion. Another gene named Peroxisome Proliferator-activated Receptor-gamma Gene (PPARG) is involved in T2DM and contributes to the 20% risk incidence when arginine replaces the proline at location 12. Both IRS1 and IRS2 polymorphism interfere with insulin signaling pathways and reduce insulin sensitivity [58]. Missense polymorphism in KCNJ11 alters insulin secretion. SLC30A8 has shown to be widely involved in Type 1 diabetes progression preliminary, and its consistent overexpression ends with the incidence of Type 2 diabetes. Since diabetes is often associated with obesity, the genes related to obesity, such as FTO and MC4R, are often connected with diabetes. Any changes in Pdx1 and Glut4 genes’ functions predominantly play a vital role in developing Type 1 diabetes [59]. Epigenetic alterations such as histone methylation, acetylation, phosphorylation, DNA methylation, chromatin remodeling, and oxidative stress play an essential role in promoting and progressing diabetic mellitus. It has been proved that the diet has a predominant control over the epigenetic factors that affect the progression of diabetes. In addition, a baby’s gestational age can influence the incidence of obesity and diabetes together [60].

### 4.2. Plant Sterols and Anti-Cholesterol Activity

The human body requires the waxy substance cholesterol to build healthy cells and synthesize hormones and digestive fluids. Overall, cholesterol within the required range helps our body to function correctly. An imbalance between the two forms of cholesterol, such as low and high lipoproteins, increases the chances of developing heart disease and stroke. The hypocholesterolemic effect of plant sterols was identified in early 1950 [61]. Still, many studies are in line to identify the molecular mechanism behind the anti-cholesterol effect of PS. So far, many studies emphasize that the plant sterol could regulate the levels of lipid variables such as LDL, HDL, triglycerides, and apolipoprotein B effectively [62]., Many studies have supported the lipid regulatory action of plant sterols; however, a lack of understanding of the mechanism prevents them from being used in clinical trials. Many reports have suggested that the plant sterols and stanols could reduce LDL levels and exert their action in a dose-dependent way [63,64]. Some data also show that the stanols could exert a dose-dependent hypocholesterolemic effect compared to sterols [65]. The efficiency of plant sterol may largely depend on the baseline levels of plasma lipids. Studies have shown that plant sterols could effectively lower the concentration of triacylglycerols and thereby reduce the synthesis of very-low-density lipoproteins. Conversely, a few detailed research studies have highlighted the negative association of phyto compounds with hypercholesterolemia impact [66,67].

### 4.3. Plant Sterols and Anti-Diabetic Effects

Plant-based medicines are commonly used to prevent many chronic diseases. They have been in use as a complementary or alternative therapy form since ancient times. For example, the anti-diabetic drug metformin, a derivative from the plant *Galega officinalis*, is widely used to prevent diabetes [68,69]. The other medicinal plants with anti-diabetic properties include *Aloe vera*, Jamun/Indian blackberry, gurmar, bitter guard, basil, yacon, fenugreek, etc. The various parts of the plants, such as leaves, seeds, roots, and fruits are used to treat diabetes [70,71,72,73,74,75]. These medicinal plants regulate many functions such as insulin secretion, insulin resistance, glucose absorption, and regulation (Table 2).

Results from animal studies have supported the potent hypoglycemic and hypocholesterolemic effects of plant sterols [76,77,78,79]. However, so far, no clinical research has been conducted to verify the impact of plant products as conventional drugs for diabetic management. The plant sterols could regulate the expression of genes such as glucose-6-phosphatase, phosphoenolpyruvate carboxykinase, and peroxisome proliferator-activated receptor-alpha and influence the rate of metabolism [80]. Misawa et al. reported that the ethanolic extract of *Aloe vera* could increase the production of insulin in Zucker diabetic fatty rats [81]. Research by Patil et al. proved the insulin-secreting efficiency of plant sterols present in cumin oil, cumin aldehyde, and cuminol with the help of the diabetic rat model [82]. The research identified that the plant sterol present in black cumin (*N. sativa*) blocks the sodium-dependent passage of glucose across the jejunum. Long-term treatment of *N. sativa* modulates glucose tolerance and body-weight reduction as equivalent to metformin in a rat model [83]. Indian satinwood (*Chloroxylon swietenia)* shows a significant hypoglycemic effect in streptozotocin-induced diabetic rats [84]. The ethyl acetate extract of fruits of weeping forsythia could effectively enhance the plasma level of insulin [85]. The ethanolic extract of scarlet gourds (*Coccinia grandis)* leaves exerts its hypoglycemic activity by reducing the plasma glucose level and increasing the serum insulin level [86]. These plant compounds are noticed for their ability to induce hypoglycemic impact via improving the mechanism of insulin secretion.

Research using the common nettle (*Urtica dioica*) leaves extract identified the gender-biased working modulation of plant sterols. The study proved that the compound could balance hypoglycemic activities in male Wistar rats. It could effectively reduce the plasma glucose level and fasting insulin resistance index in male rats [87]. The extract of cashew tree (*Anacardium occidentale)* could significantly reduce the blood glucose levels in STZ induced diabetic rats. In this experiment, the compound showed its ability to reduce fasting glucose levels [88]. An oil extracted from garlic (*Allium sativum)* helps to improve insulin secretion and glucose tolerance. It proved that the extract could enhance glycogenesis by increasing GLUT4 expression in the rat model [89]. The bark extract of the Sapphire berry family (*Symplcos cochinchinesis)* has been shown to improve insulin resistance and thus reduce glucose levels [90]. Similar results are observed upon the use of the extract of *Helicteres angustifolia* [91]. Other plant extracts that work by a similar mechanism to reduce the blood glucose level with effective insulin sensitivity modulation include the *Pleurotus ostreatus*, *Afzelia africana*, *Uvaria chamae*, and resveratrol [92,93,94,95].

Some phyto compounds act by inhibiting the α-glucosidase, thereby preventing carbohydrate metabolism. Reduced carbohydrate metabolism results in a decreased rate of glucose assimilation and could lead to a reduced postprandial glucose level in the blood. Many plants such as basil contain phyto compounds that inhibit enzymes such as α- glucosidase and α-amylase, thereby acting as a potent anti-hyperglycemic agent [96]. On chromatographic separation, the basil leaves are found to contain flavonoids, glycosides, steroids, and tannins [97]. The polyphenol compounds isolated from the jute plant exert its potential by inhibiting ACE and lowering the levels of α- glucosidase and α- amylase significantly [98,99]. Certain legumes like soybean have been shown to contain polyphenolic compounds such as isoflavones [100]. The phyto compounds of fig fruits such as vitexin and isovitexin were shown to have glucose reduction properties in sucrose-loaded mice [101]. The figs (*Ficus deltoidea)* are considered an alternative natural remedy for diabetes and are also available in many commercial forms. Plant sterols have been shown to improve insulin production, GLUT4 expression, and insulin sensitivity. As well it inhibits α glucosidase, α amylase, and ACE functions. In addition, the plant-derived compounds also regulate genetic and epigenetic factors to counteract diabetes. More clinical research is necessary to support the therapeutic potential of plant sterols in preventing diabetes.

**Table 2:** Anti-diabetic activity of different herbs.

Botanical Name	Common Name	Components Used	Animal Studies	Effects	Reference
***Aloe barbadensis***	*Aloe vera*	Leaves	Diabetic rats	Significant reduction in the levels of the enzymes that facilitate carbohydrate metabolism	[81]
***Cuminum cyminum***	Cumin	seeds	Diabetic rats	Improves insulin secretion	[82]
***Nigella sativum***	Black cumin	Seeds	Rats	Improves glucose tolerance	[83]
***Chloroxylon switenia***	Indian satinwood	Barks	Diabetic albino rats	Decreases blood glucose level	[84]
***Forsythia suspense***	Weeping forsythia	Fruits	STZ induced Kunming mice	Significant reduction in blood glucose level	[85]
***Coccinia grandis***	Scarlet gourds	Leaf	Diabetic Wistar rats	Improves insulin-secretagogue and cytoprotective activities	[86]
***Afzelia africana***	African mahogany	Stem	Diabetic Wistar rats	Reduces hyperglycemia	[93]
***Urtica dioica***	Common nettle	Leaf	Fructose induced Insulin resistance Wistar rats	Significantly reduces hyperglycemia and insulin resistance	[87]
***Anacardium accidentale***	Cashew tree	Leaf	Diabetes induced female albino Wistar rats	Significant reduction in the levels of serum glucose, glycosylated haemoglobin, FIRI, and serum insulin	[88]
***Pleurotus ostreatus***	Oyster mushroom		Diabetes induced male Wistar rats	Significant reduction in blood glucose level	[92]
***Uvaria chamae***	Bush banana	Root	Diabetes induced albino rats	Significant improvement in the regeneration of islets of Langerhans	[94]
***Cinnamomum zeylanicum***	Cinnamon	Bark	STZ-induced rats	Significantly diminishes α-glucosidase activity	[73]
***Ocimum basilicum***	Basil	Leaves		Significantly inhibits α amylase activity in a dose-dependent manner	[97]
***Corchorus olitorius***	Jute	Leaves		Significantly inhibits the enzymatic activities of α-amylase, α-glucosidase, and ACE	[99]
***Ficus deltoidea***	Fig	Leaves, Flowers	STZ-induced diabetic rats	Significantly lowers the blood glucose level	[101]
***Holarrhena antidysenterica***	Bitter oleander	Seeds	Starch-loaded normoglycemic rats	Interferes with starch digestion	[75]
***Olea europaea***	Olive	Leaves	STZ-induced diabetic rats	Inhibits α amylase activity	[72,74]
***Glycine max***	Soybean	Soybean		Significantly lowers the levels of α-amylase, α- glucosidase and ACE	[100]

## 5. Conclusions

Phyto compounds, the naturally obtained formulations from plants, are well known for their effective anti-diabetic properties. They can exert their function directly by interacting with several signaling pathways associated with diabetes. They also have a potent hypocholesterolemic effect, which could indirectly counteract those obesity-related issues connected with diabetes. In conclusion, we strongly support that due to fewer side effects with significant medicinal character, plant sterols and stanols can be widely used to prevent diabetes. More clinical trials are needed to reveal the anti-diabetic benefits of plant compounds to commercialize them as therapeutic agents.

## Figures and Tables

**Table 1:** Represents the list of plant-derived foods, their scientific names, and the volume of their phytosterols.

Plant-Derived Foods	Scientific Names	Total PS (mg/100 g)	Reference
Zucchini	*Cucurbita pepo* L.	0.63	[18]
Eggplant	*Solanum melongena* L.	0.5	[18]
Broccoli	*Brassica oleracea* var. Italica	4–50	[18]
Carrot	*Daucus carota* L.	16–30	[18]
Cauliflower	*Brassica oleracea* var. *Botrytis* L.	44	[18]
Endive	*Cichorium endıvia* L.	16–20	[19]
Spinach	*Tetragonia expansa*	16	[19]
White cabbage	*Brassica oleracea* var. Capital	27.4	[19]
Tomato	*Lycopersicum esculentum* Mill.	9–10	[19]
Green bean	*Phaseolus vulgaris*	10–12	[19]
Brown rice	*Oryza sativa*	18–20	[19]
Polished rice	*Oryza sativa*	9–10	[19]
Green pea	*Pisum sativum*	25	[19]
Brown beans	*Phaseolus vulgaris* L.	16	[20]
Black beans	*Phaseolus vulgaris* L.	15	[20]
Chickpeas	*Cicer arietinum*	120	[20]
Lentil	*Lens esculenta*	117	[20]
Soybean	*Glycine max* L. Merr.	32–35	[20]
Black soybean	*Glycine max* L. Merr.	17–18	[21]
Linseed	*Linum usitatissimum*	44–45	[21]
Acai	*Euterpe oleracea*	14	[21]
Avocado	*Persea americana*	75	[21]
Pineapple	*Ananas comosus* L. Merril	3–5	[21]
Banana	*Musa acuminata* × *Musa balbisiana*	12–16	[21]
Coconut	*Cocos nucifera*	14	[22]
Guava	*Psidium guajava* L.	3–5	[22]
Orange	*Citrus sinensis*	23–24	[22]
Apple	*Malus Domestica*	13–18	[22]
Papaya	*Carica papaya*	4–5	[22]
Mango	*Mangifera indica* L.	1–2	[22]
Strawberry	*Fragaria vesca* L.	10–15	[22]
Canola (Rapeseed)	*Brassica napus*	250–878	[22]
Coconut	*Cocos nucifera*	73–75	[22]
Sunflower	*Helianthus annuus*	400–500	[22]
Corn	*Zea mays*	686–1400	[22]
Soybean	*Glycine max*	203–328	[22]
Olive	*Olea europaea*	114–162	[22]
